# The chicken cecal microbiome alters bile acids and riboflavin metabolism that correlate with intramuscular fat content

**DOI:** 10.3389/fmicb.2024.1494139

**Published:** 2024-12-10

**Authors:** Xiaoxia Long, Fuping Zhang, Liqi Wang, Zhong Wang

**Affiliations:** Key Laboratory of Animal Genetics, Breeding and Reproduction in the Plateau Mountainous Region, Ministry of Education, College of Animal Sciences, Guizhou University, Guiyang, China

**Keywords:** chickens, intramuscular fat, cecal microbiota, metabolomics, integrative omics

## Abstract

Intramuscular fat (IMF) is a key indicator of chicken meat quality and emerging studies have indicated that the gut microbiome plays a key role in animal fat deposition. However, the potential metabolic mechanism of gut microbiota affecting chicken IMF is still unclear. Fifty-one broiler chickens were collected to identify key cecal bacteria and serum metabolites related to chicken IMF and to explore possible metabolic mechanisms. The results showed that the IMF range of breast muscle of Guizhou local chicken was 1.65 to 4.59%. The complexity and stability of ecological network of cecal microbiota in low-IMF chickens were higher than those in high-IMF chickens. Cecal bacteria positively related to IMF were *Alistipes*, *Synergistes* and *Subdoligranulum*, and negatively related to IMF were *Eubacterium_brachy_*group, *unclassified_f_Lachnospiraceae*, *unclassified_f_Coriobacteriaceae*, *GCA-900066575*, *Faecalicoccus*, and so on. Bile acids, phosphatidylethanolamine (Pe) 32:1 and other metabolites were enriched in sera of high-IMF chickens versus low-IMF chickens while riboflavin was enriched in sera of low-IMF chickens. Correlation analysis indicated that specific bacteria including *Alistipes* promote deposition of IMF in chickens via bile acids while the *Eubacterium_brachy* group, and *Coriobacteriaceae* promoted formation of riboflavin, glufosinate, C10-dats (tentative), and cilastatin and were not conducive to the IMF deposition.

## Introduction

Significant improvements in broiler chicken weights, growth and feed conversion have relied on genetic selection of these particular phenotypes but these gains have resulted in a decrease in meat quality (Tian et al., 2021). Hence, improving meat quality has become an urgent problem for breeders. Intramuscular fat (IMF) is a key trait that defines meat quality and refers to fat deposition between muscle fibers or within muscle cells (Fang et al., 2017). IMF content affects the sensory qualities of meat including flavor, tenderness and juiciness (Hirai et al., 2023; Li et al., 2022). In addition, the genetic basis of IMF has low heritability (0.11–0.18) (Chabault et al., 2012; Chen et al., 2008; Jiang et al., 2017) indicating environmental factors play significant roles in IMF.

Numerous studies have confirmed that the gut microbiota plays an important role in host fat deposition (Xie et al., 2022; Chen et al., 2022; Wen et al., 2023) as well as modulation of muscle function and cognition (Jing et al., 2021; Lei et al., 2022). Microbiota composition of the chicken duodenum and cecum could explain 24 and 21% of the variation in abdominal fat mass, respectively after correcting for host genetic effects (Wen et al., 2019). Some gut bacteria linked to abdominal fat deposition have been identified and include *Olsenella*, *Slackia*, and *Methanobrevibacter* that promote while *Bacteriodes salanitronis*, *Bacteriodes fragilis*, and *Parabacteriodes distasonis* that inhibit fat deposition in chickens (Wen et al., 2023, 2019; Xiang et al., 2021; Zhang et al., 2022). There have also been attempts to modulate the fat deposition process through targeted intervention of gut microbiota in chickens. For example, feeding the probiotic mixtures of *Clostridium butyricum* (Yang et al., 2010), *Lactobacillus farciminis* and *Lactobacillus rhamnosus* (Eglite et al., 2023) to chickens could increase polyunsaturated fatty acid content in muscle. A new study of fecal microbiota transplantation related to the promotion of abdominal fat deposition indicated roles for *Lachnoclostridium* and *Christensenellaceae_R-7_* group (Lei et al., 2022). These studies have proven the close relationship between gut microbiota and fat deposition and indicated a great potential for improving IMF in broilers by regulating gut microbiota. However, researchers found that the composition and formation mechanisms of intramuscular fat and abdominal fat differ (Zerehdaran et al., 2004) and there are few studies on the impact of gut microbiota on IMF of chickens. Only a study have been linked the cecal microbiota ecosystems to high IMF deposition in broilers, and found that the lower abundance of cecal *vadinBE97* was related to higher IMF levels in muscle tissues (Wen et al., 2023).

Although previous studies have initially revealed the gut microbiota ecosystem, enterotypes and bacteria that are linked to IMF (Wen et al., 2023), there is little data on the metabolites and signaling molecules regulated by IMF in chickens. Studies in humans or other animals have identified that metabolites produced by gut microbiota including fatty acids (Lahiri et al., 2019), bile acids (Mancin et al., 2023) and branched-chain amino acids (Jang et al., 2016) were important intermediate substances in regulating host fat deposition (Krause et al., 2020; Wen et al., 2023). The gut microbiota utilized these metabolites as substrates or signals to activate receptors and affect metabolic processes related to muscle fat. Myristic acid, heptadecanoic acid and trans-monounsaturated fatty acids have been linked to IMF content in lambs (Realini et al., 2021). Diet supplements containing galacto- and xylo-oligosaccharides can regulate the composition of cecal microbiota, affect metabolism processes and thereby regulate IMF in chickens (Yang et al., 2022). However, the metabolites related to IMF have been incompletely described.

With the application of metabolomics technology, researchers can detect thousands of metabolites simultaneously making it possible to trace metabolic mechanisms of complex traits (Wu et al., 2018). Hence, we propose a hypothesis that specific gut bacteria will regulate fat metabolism processes via metabolites in chickens and thereby affect IMF formation. To verify this, we collected the samples from Guizhou local chickens and measured their breast muscle IMF content. We integrated microbial 16S rRNA gene sequencing and non-targeted metabolomics technology to identify key bacteria and serum metabolites related to IMF, and to explore the metabolic mechanisms used by cecal microbiota that affect IMF formation. The results offer new insights into the formation of IMF and provide a research basis for development of probiotics/prebiotics to increase IMF deposition in chickens.

## Materials and methods

### Animals and sample collection

The chickens used in this study were raised at the research farm of Guizhou University from June 2022 to October 2022. A total of 51 Guizhou yellow chickens (25 males and 26 females) were collected. All chickens had the same batches, feeds, chicken houses and feeding methods. Specifically, chickens were raised with three-stair iron cages in a same house. The stocking density were: 16 chickens from 0 to 4 weeks (male and female mixed); 8 from 4 to 10 weeks (4 males and 4 female mixed); one in a cage from 10 to 18 weeks. The chicken house had no temperature control system and used roller shutters to regulate ventilation and temperature. The daily lighting time was 16 h. The temperature of the chicken house during the experiment was 15–35°C. In accordance with the health and epidemic prevention requirements of chicken farms, the chicken house was cleaned and disinfected every week. The feed was obtained commercially and chickens were fed twice a day in the morning and afternoon and had free access to feed and water. The nutritional composition is shown in Supplementary Table S1. All chickens were vaccinated according to routine immunization procedures to Marek’s disease, Newcastle disease, infectious bronchitis, bursal virus and avian influenza. The chickens were weighed every 2 weeks. No antibiotic or probiotics were added to the feed within 1 month prior to sample collection.

**Figure 1:**
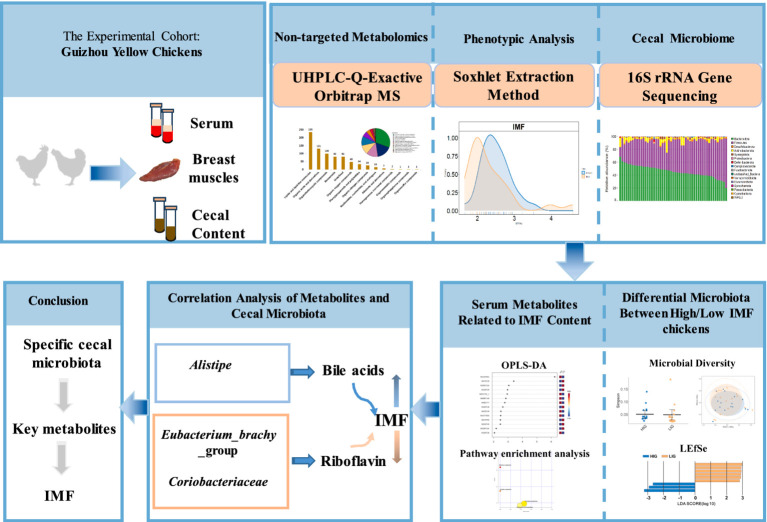
Experimental flow chart. A total of 51 Guizhou yellow chickens were collected. The intramuscular fat (IMF) content of breast muscle of all chickens was measured. Cecal contents and serum samples were collected for microbial 16S rRNA gene sequencing and non-targeted metabolome detection, respectively. High- and low-IMF chicken groups were established in the experimental cohorts. The differences in the cecal microbiota and metabolome between two groups were compared and the correlation analysis of differential microbes and differential metabolites was conducted.

At the age of 18 weeks, blood was collected from the chicken wing vein and allowed to stand for 1–2 h to separate the serum. The chickens were then euthanized by CO_2_ anesthesia and asphyxiation (Haetinger et al., 2021). Then ipsilateral distal cecum of each chicken was cut open with scissors that had been disinfected with alcohol. About 2 g of cecum contents were squeezed out and were collect into an Eppendorf tube and placed in liquid nitrogen. The whole breast muscle was excised from the right of each chicken and surface fat was removed and discarded. Then all the collected serum, cecum contents samples, and breast muscle samples were stored at −80°C refrigerator. Cecal content samples and serum samples were sent to Majorbio Bio Pharm Technology (Shanghai, China) and Shanghai Applied Protein Technology (Shanghai, China) on dry ice for microbial 16S rRNA gene sequencing and non-targeted metabolome detection, respectively. The experimental flow chart is shown in Figure 1.

### IMF measurements

The IMF content of breast muscle was measured using Soxhlet extraction method as reported previously (Chang and Lei, 2010). Specifically, 20 g of breast muscle was minced with a meat grinder, dehydrated and dried. The dried samples were ground into powder, transferred to a cartridge filter and extracted with a Soxhlet extraction device using anhydrous ether at 30–60°C for 65 min. The liquid extract was concentrated under reduced pressure and the residue was dried in a forced air oven at 80°C for 8 h until constant mass. Three replicates were set for each sample and the average value was considered as final value of IMF content. IMF content = (total weight of meat and filter paper after drying − total weight of meat and filter paper after extraction)/weight of fresh meat × 100%.

Eight male and eight female chickens that possessed the highest IMF content were selected to construct a high-IMF group (HIG, *n* = 16) and eight male and eight female chickens with the lowest IMF content were selected to construct low-IMF group (LIG, *n* = 16, Supplementary Table S2).

### Cecal microbiota DNA extraction, V3–V4 region sequencing of 16S rRNA gene and data processing

Total DNA from cecal samples was extracted using the Magnetic Soil and Stool DNA Kit (Tiangen, Beijing, China) according to the manufacturer’s protocol. DNA concentrations and quality were determined by a Nanodrop-1000 (Thermo Fisher, Waltham, MA, United States) and 0.8% agarose gel electrophoresis. The V3–V4 region of 16S rRNA gene were amplified using the fusion primers 338F (5′-ACTCCTACGGGAGGCAGCAG-3′) and 806R (5′-GGACTACHVGGG TWTCTAAT-3′) under the annealing temperature of 55°C with 27 cycles. The PCR amplicons were purified with agarose gel by an AxyPrep DNA Gel Extraction Kit (Axygen, Union city, CA, United States).

Amplicon libraries were sequenced on an Illumina MiSeq platform (Illumina, San Diego, CA, United States) with a paired-end strategy. Quality control of the raw data was performed to filter the barcode, primer, low-quality and high nucleotide ambiguities sequences with custom scripts. FLASH (v.1.2.11) was used to assemble the paired-end clean reads into tags (Magoc and Salzberg, 2011). High-quality tags were clustered into Amplicon Sequence Variants (ASVs) using DADA2 (Callahan et al., 2016). RDP classifier program (v2.2) (Wang et al., 2007) was applied to assign ASVs based on 16S rRNA gene sequences. The representative sequence of each ASV was screened for further annotation by Silva database (Release 132, http://www.arb-silva.de) (Quast et al., 2013). The α-diversity of microbiota via the Chao1, Shannon and phylogenetic diversity (PD) indices was analyzed using QIIME2 (Bolyen et al., 2019). Principal coordinate analysis (PCoA) based on Bray-Curtis distance was used to evaluate the β-diversity of gut microbial community.

### Non-targeted metabolomics detection for serum samples

#### Extraction of serum samples

After samples thawed slowly at 4°C, 100 μL serum were added 300 μL pre-cooled methanol/acetonitrile/water solution (2:2:1, v/v), vortexed and ultrasonicated at low temperature for 30 min and then stand 10 min at −20°C. Samples were then centrifuged at 14,000 × g for 20 min at 4°C and the supernatant was dried under vacuum. The samples were then dissolved with 100 μL acetonitrile/water solution (1:1, v/v) for analysis.

#### Analytical conditions for LC-MS

The UHPLC-Q-Exactive Orbitrap MS mass spectrometry was used to perform non-targeted metabolomics detection on serum samples (Cai et al., 2015; Dunn et al., 2011; Wang et al., 2016). The ultra-high performance liquid chromatography system (UHPLC) (Thermo Fisher Scientific, Pittsburg, PA, United States) was equipped with a LC BEH Amide column (2.1 mm × 100 mm, 1.7 μm). The mass spectrometer detector was Tandem Orbitrap MS Q Exactive HFX (Thermo Fisher Scientific, Pittsburg, PA, United States).

##### Chromatographic conditions

Sample components were separated using an Agilent 1290 Infinity HILIC column. The column temperature was set at 25°C, the flow rate was 0.5 mL/min and the injection volume was 2 μL. Mobile phase A: water /25 mM ammonium acetate/25 mM ammonia water, mobile phase B: acetonitrile. The gradient elution program was as follows: 0–0.5 min, 95% B; 0.5–7.0 min, B changed linearly from 95 to 65%; 7.0–8.0 min, B changed linearly from 65 to 40%; 8.0–9.0 min, B was maintained at 40%; 9.0–9.1 min, B changed from 40 to 95%; 9.1–12.0 min, B was maintained at 95%. During the analysis process, the samples were placed in the autosampler at 4°C. In order to avoid the influence caused by the fluctuation of the instrument detection signal, samples were analyzed continuously in random order. QC samples were inserted into the queue to monitor and evaluate the stability of the system and the reliability of experimental data.

##### Mass spectrometry conditions

An AB Triple TOF 6600 mass spectrometer was used to collect primary and secondary spectra of samples. After samples were separated by UHPLC, mass spectrometry analysis was performed with a Triple TOF 6600 mass spectrometer (AB SCIEX), and electrospray ionization (ESI) positive ion and negative ion modes were used for detection. The ESI source parameters were as follows: atomization gas auxiliary heating gas 1 (Gas1): 60, auxiliary heating gas 2 (Gas2): 60, curtain gas (CUR): 30 psi, ion source temperature: 600°C, spray voltage (ISVF) ± 5,500 V (positive and negative modes); primary mass-to-charge ratio (m/z) detection range: 60–1,000 Da, scanning accumulation time: 0.20 s/spectra; secondary ion m/z ratio detection range: 25–1,000 Da, scan accumulation time was 0.05 s/spectra. The secondary mass spectrum was obtained using data-dependent acquisition mode (IDA) using peak screening. Declustering voltage (DP): ±60 V (positive and negative modes), collision energy: 35 ± 15 eV, IDA parameters were as follows: dynamic exclusion isotope ion range: 4 Da, each scan collected 10 fragment spectra.

#### Metabolome data preprocessing

ProteoWizard MSConvert was applied to convert raw MS data to MzXML files and then imported into XCMS software (Tautenhahn et al., 2012). Parameters for peak selection were: centWave m/z = 10 ppm, peak width = c (10, 60), prefilter = c (10, 100). For peak grouping: bw = 5, mzwid = 0.025, minfrac = 0.5. Isotope and adduct annotation were conducted by CAMERA (Collection of Metabolite Profile Annotation Algorithms). Variables with no less than 50% non-zero readings in at least one group were kept in the extracted ion features. Metabolite compounds were identified though comparing MS/MS spectra and accurate m/z values (<10 ppm) with an internal database supplied with the instrument.

### Cytokines detection

INF-γ, IL-1β, IL-5, IL-6, IL-17, and IL-22 were measured using commercial ELISA kits (Ziker Biological Technology, Shenzhen, China) according to the manufacturer’s protocols. The detection limits were 5 pg/mL (IFN-γ), 40 pg/mL (IL-1β), 5 pg/mL (IL-5), 2 pg/mL (IL-6), 3 pg/mL (IL-17), and 2 pg/mL (IL-22).

### Statistical analysis

#### Construction of cecal microbiota co-occurrence network

The SparCC algorithm (Friedman and Alm, 2012) was used to construct a microbial co-occurrence network. The correlation between ASVs was calculated based on relative abundance using the PCIT algorithm (Reverter and Chan, 2008). The correlation coefficients between two ASVs (nodes) were calculated with an absolute sparse correlation coefficient and paired taxa with a correlation coefficient >0.45 were retained to construct the co-occurrence network. Cytoscape (3.7.1) (Lopes et al., 2010) was employed to visualize the co-occurrence network and calculate the network topological characteristics including clustering coefficient, density and scale-free properties. The stability of the co-occurrence network was represented by the proportion of negative correlations (competitiveness) to the total number of correlations (Coyte et al., 2015; Hernandez et al., 2021). The complexity of a co-occurrence network was represented by the average number of lines connected to each point (Bader and Hogue, 2003).

#### Statistical analysis of metabolome data

The metabolite data of peak area was normalized using Log_10_ conversions. The processed data was analyzed using the online platform MetaAnalyst 6.0.^1^ Differential metabolites were identified using the orthogonal partial least squares-discriminant analysis (OPLS-DA). The reliability of the model was assessed using 7-fold cross-validation and response permutation tests. Variable projection (VIP) values were calculated for each variable in the model to indicate its contribution to the classification. Metabolic pathway analysis (MetPA) was used to evaluate the interactions between metabolites and to reveal the importance of metabolic pathways. If the impact value exceeds 0.10, the metabolic pathway was important.

#### Other statistical analyses

Differential cecal microbiota at the plylum and ASV levels were identified using LEfSe (Linear discriminant analysis with effect size estimation)^2^ (Segata et al., 2011) with LDA >2.0 and *p* < 0.05. Spearman correlation analysis was used to identify relationships between cecal microbiota and serum metabolites. And the correlation among IMF, body weight, and cytokine content were explored by spearman correlation analysis as well. *p* < 0.05 indicates a significant correlation, while the absolute value of correlation coefficient was used to represents the magnitude of the correlation. |*r*| < 0.2 indicates no correlation, 0.21 < |*r*| < 0.40 indicates a weak correlation, 0.41 < |*r*| < 0.60 indicates a moderate correlation, and |*r*| > 0.61 indicates a strong correlation. Wilcoxon test was used to compare the α-diversity of cecal microbiota between the HIG and LIG groups. Visualization of results and other statistical analyses were performed using R (R Core Team, 2022).

## Results

### The IMF content of breast muscles

**Figure 2:**
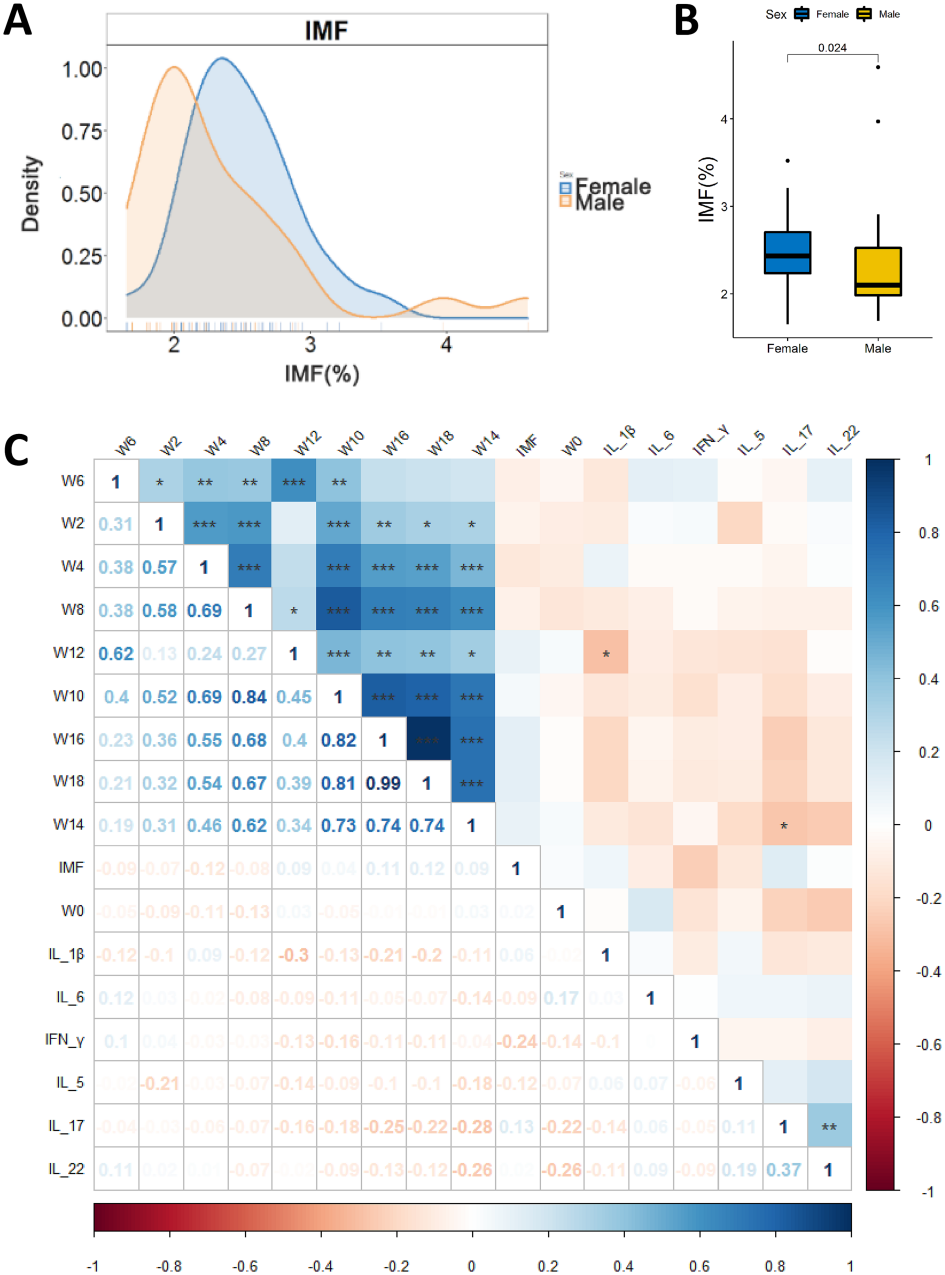
Relationships between IMF content in chicken breast muscle and gender, body weight and serum cytokines. **(A)** Distribution of IMF content of male and female chickens (25 males and 26 female). *y*-axis represents frequency of IMF level; *x*-axis represents IMF %. **(B)** Comparison of IMF content in breast muscle of male and female chickens. **(C)** Correlations of IMF content of breast muscle, body weight and serum cytokine levels. ^*^p < 0.05, ^**^p < 0.01, and ^***^p < 0.001.

The IMF content of breast muscles of the experimental chickens ranged from 1.65 to 4.59% (Figure 2A and Supplementary Table S3). Gender have significant effects on IMF (*p* = 0.024, Wilcoxon test, Figure 2B). We performed Spearman correlation analysis on IMF content and body weights across the time-span of the experiments (Figure 2C), and it was found that there was no significant correlation between body weights and IMF content of chicken. High-IMF group (HIG, *n* = 16) and low-IMF group (LIG, *n* = 16) were constructed based on IMF content of chickens. Student’s t test was used to compare the body weight of chickens at different ages. No significant difference in body weight was found between HIG and HIG at 0, 2, 4, 6, 8, 10, 12, 14, 16, and 18 weeks of age (Table 1). In addition, since previous studies have linked fat deposition in pigs and humans to serum cytokine levels (Chen et al., 2021; Wedell-Neergaard et al., 2019), we measured INF-*γ*, IL-1β, IL-5, IL-6, IL-17, and IL-22 in serum samples, and no significant correlations were found between cytokine levels and IMF content (Figure 2C).

**Table 1:** Comparison of the weights of chickens at different weeks between HIG and LIG.

Weeks	HIG	LIG	*p*-value	Weeks	HIG	LIG	*p*-value
0	36.04 ± 7.17	35.16 ± 6.66	0.72	10	1153 ± 179.29	1141.25 ± 125.50	0.83
2	155.93 ± 27.49	156.83 ± 20.87	0.92	12	1407.81 ± 183.58	1422.31 ± 368.07	0.89
4	330.94 ± 54.54	351.06 ± 64.77	0.34	14	1684.81 ± 536.38	1532.25 ± 199.17	0.29
6	562.63 ± 108.89	644.06 ± 144.01	0.08	16	1921.44 ± 294.29	1829.75 ± 233.31	0.33
8	893.16 ± 146.09	910.94 ± 107.39	0.69	18	2036.69 ± 311.98	1922.88 ± 273.42	0.28

HIG, chickens with high intramuscular fat content (*n* = 16); LIG, chickens with low intramuscular fat content (*n* = 16).

### Differences of cecal microbiota between high/low-IMF chickens

**Figure 3:**
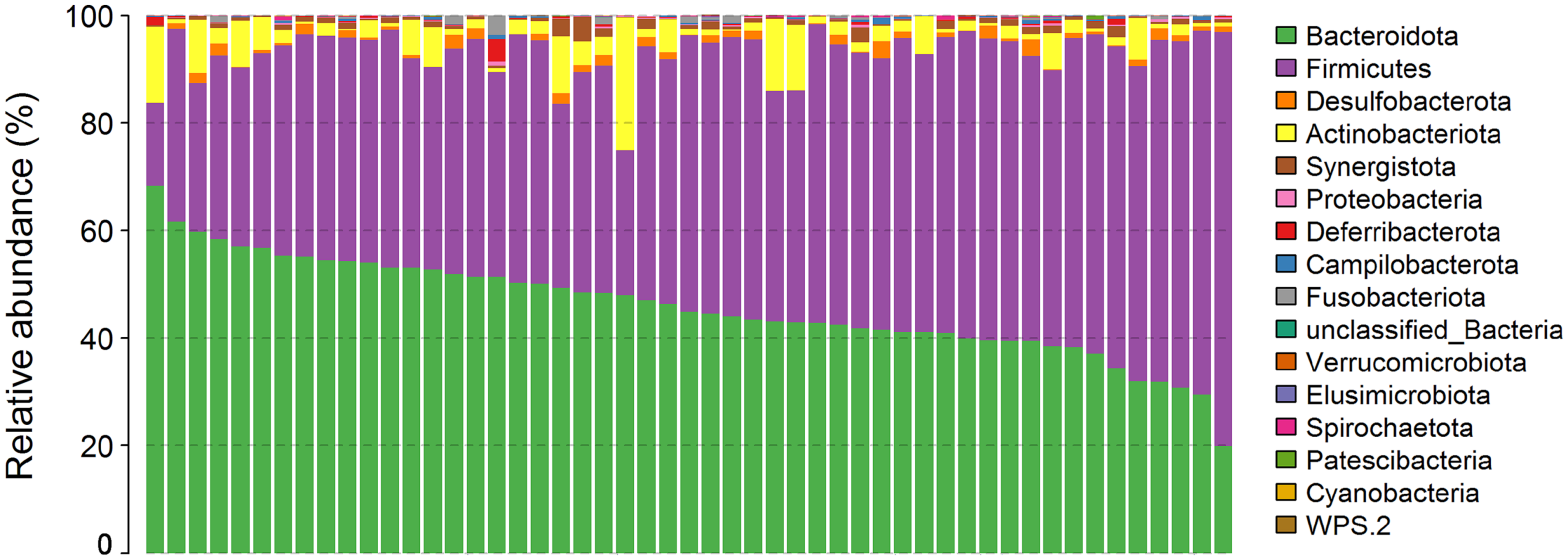
The profiles of microbial compositions of cecum at the phylum level. The samples were ordered following the abundance of Bacteroidetes.

**Figure 4:**
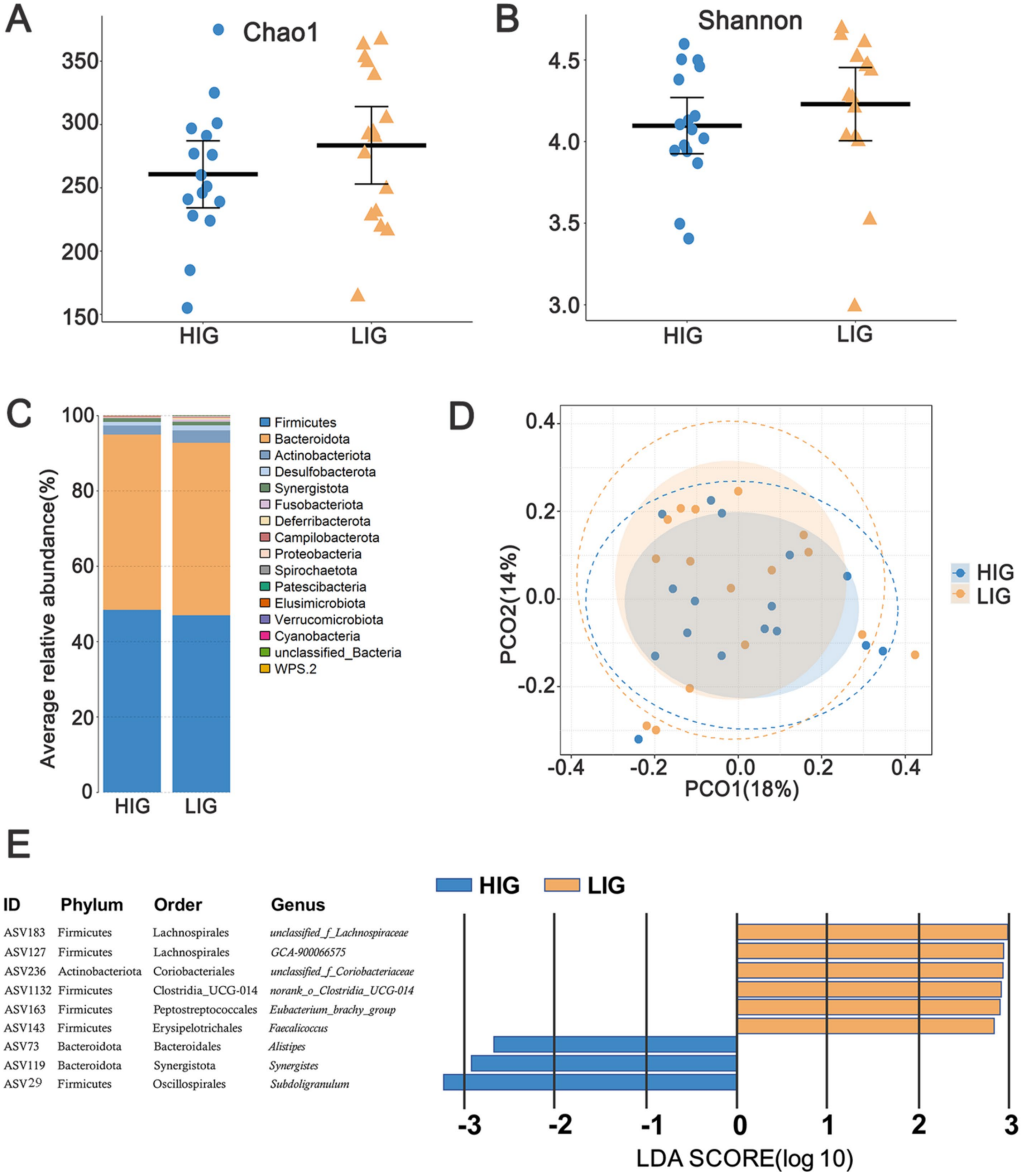
The difference of diversity and composition of cecal microbiota between HIG and LIG chickens. Comparison of the α-diversity of cecal microbiota between HIG (*n* = 16) and LIG (*n* = 16) with **(A)** Chao1 index, and **(B)** Shannon index. **(C)** Average relative abundance of cecal microbiota at the phylum level in HIC and LIG chickens; **(D)** PCoA based on Bray–Curtis distance showing the cecal microbiota composition between the two groups. **(E)** Nine ASVs showing significantly different relative abundance between HIG and LIG. PCoA, Principal coordinate analysis; HIG, chickens with high intramuscular fat content; LIG, chickens with low intramuscular fat content.

A total of 15 bacterial phyla were annotated in the cecal microbiota of these chickens. The relative abundance of Bacteroidetes (45.90%), Firmicutes (47.16%), and Actinobacteriota (4.01%) was >1% (Figure 3). By comparing the diversity and composition of the cecal microbiota of HIG and LIG chickens, it was found that there was no significant difference in the diversity (Figures 4A,B,D) and the composition at the phylum level (Figure 4C) of cecal microbiota between the two groups.

In order to explore the differences of structural characteristics of cecal microbiota networks between the two groups, we screened out those ASVs with relative abundance >0.05% to construct a co-occurrence network. After quality control, 242 and 245 ASVs were obtained from cecal microbiota of HIG and LIG, respectively. The results showed that the stability index of cecal microbiota network of LIG was 49.16% and HIG was 48.56%, and the complexity indices for LIG and HIG were 9.54 and 7.87, respectively, indicating a more stable and complex microbiota network in the cecum of the LIG than in HIG (Table 2).

**Table 2:** Comparison of structural characteristics of cecal microbiota co-occurrence network between HIG and LIG chickens.

Group	HIG	LIG
Nodes	242	245
Edges	1,905	2,339
Negative edges	925	1,150
Complexity	7.87	9.54
Stability (%)	48.56%	49.16%

The paired taxa with absolute sparse correlation coefficient >0.45 were selected for the network construction. Complexity: the average number of edges connected to each node. Stability: the proportion of negative correlations to the total number of correlations. HIG, chickens with high intramuscular fat content; LIG, chickens with low intramuscular fat content.

We further discriminated the differential ASVs between HIG and LIG using LEfSe (Supplementary Table S4). Under the thresholds of LDA >2.0 and *p* < 0.05, nine ASVs were identified with their relative abundance that significantly differed between the two groups. Six ASVs were enriched in LIG, including the Clostridia class members ASV183 (*unclassified_f_Lachnospiraceae*), ASV127 (*GCA-900066575*), ASV1132 (*norank_f_norank_o_Clostridia_UCG-014*), and ASV163 (*Eubacterium_brachy_*group). ASV143 (*Faecalicoccus*) in the Bacilli class and ASV236 (*unclassified_f_Coriobacteriaceae*) in the Coriobacteriia class were also represented. ASV73 (*Alistipes*), ASV119 (*Synergistes*) and ASV29 (*Subdoligranulum*) were enriched in HIG chickens (Figure 4E).

### Serum metabolites related to IMF content in chickens

**Figure 5:**
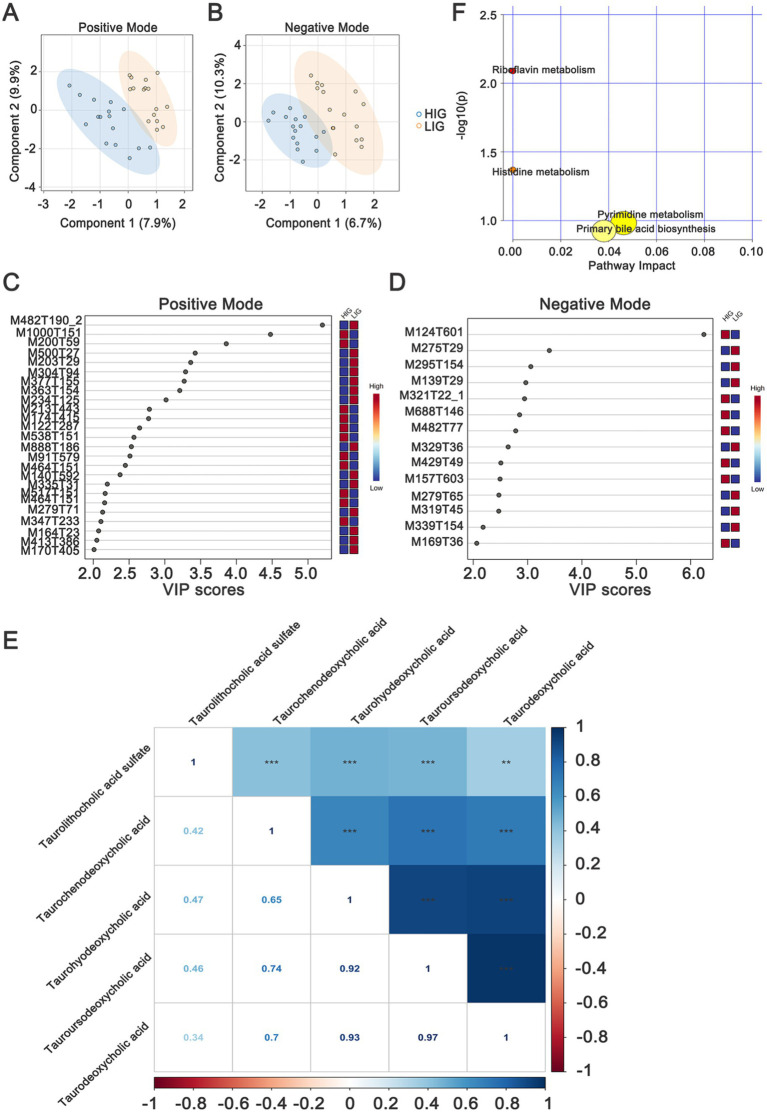
Identifying serum metabolites and metabolic pathways that differ between HIG (*n* = 16) and LIG (*n* = 16) chickens. OPLS-DA analysis of serum metabolomics profiles showed a clear separation between HIG and LIG chickens both in **(A)** positive and **(B)** negative ion modes. Variable importance in projection (VIP >2) scores for the top serum metabolites in **(C)** positive and **(D)** negative modes contributing to variation in metabolic profiles of HIG and LIG chickens. The relative abundance of metabolites is indicated by a colored scale from blue to red representing the low and high, respectively. **(E)** The correlation of five bile acids which are related with IMF. **(F)** Pathway enrichment analysis of metabolites associated with IMF in chickens. OPLS-DA, orthogonal partial least squares-discriminant analysis; HIG, chickens with high intramuscular fat content; LIG, chickens with low intramuscular fat content. The metabolites are shown in Table 3.

Non-targeted metabolomics detection was performed on serum samples and 7,086 and 4,910 metabolite features were obtained, including 494 and 272 metabolites annotated in positive and negative modes, respectively (Supplementary Table S5). An OPLS-DA model was used to identify the differential metabolites, and the results showed that samples from HIG and LIG could be well separated in both positive (Figure 5A) and negative modes (Figure 5B).

In positive mode, there were 25 differential metabolites (VIP >2, *p* < 0.05) between the two groups, in which, 11 metabolites were enriched in HIG and 14 metabolites were enriched in LIG. The most abundant metabolites enriched in HIG were bile acids, including taurodeoxycholic acid, taurochenodeoxycholic acid, taourusodeoxycholic acid and taurohyodeoxycholic acid. In addition, Arg-Gly-Asp, benzyl alcohol, benzamide, β-d-glucopyranosiduronic acid, 5-[3-[(2,2,3,3-tetramethylcyclopropyl) carbonyl]-1h-indol-1-yl] pentyl, and other some metabolites were also enriched in HIG. Riboflavin (vitamin B2) as well as some metabolites of amino acids (1-methylhistidine, NG,NG-dimethyl-l-arginine and 3-amino-2,3-dihydrobenzoic acid) were enriched in the LIG (Figure 5C and Table 3).

**Table 3:** Differential serum metabolites between HIG and LIG chickens.

Ion mode	ID	Enriched group	Metabolites
Positive mode	M91T579	HIG	Benzyl alcohol
	M517T151	HIG	Taurodeoxycholic acid
	M1000T151	HIG	Taurochenodeoxycholic acid
	M122T287	HIG	Benzamide
	M464T151	HIG	Tauroursodeoxycholic acid
	M174T415	HIG	Allidochlor
	M486T151	HIG	Beta-d-glucopyranosiduronic acid, 5-[3-[(2,2,3,3-tetramethylcyclopropyl)carbonyl]-1h-indol-1-yl]pentyl
	M200T59	HIG	6-tert-butyl-3-methylsulfanyl-2h-1,2,4-triazin-5-one
	M347T233	HIG	Arg-Gly-Asp
	M213T443	HIG	Chromone-3-carboxylic acid
	M538T151	HIG	Taurohyodeoxycholic acid
	M377T155	LIG	Riboflavin
	M170T405	LIG	1-methylhistidine
	M203T29	LIG	NG,NG-dimethyl-l-arginine
	M363T154	LIG	Glufosinate
	M140T592	LIG	3-amino-2,3-dihydrobenzoic acid
	M279T71	LIG	Dibutyl phthalate
	M164T23	LIG	1-deoxynojirimycin
	M304T94	LIG	Fenpropimorph
	M234T125	LIG	Tebutam
	M335T31	LIG	Docosatrienoic acid
	M888T186	LIG	1-stearoyl-2-arachidonoyl-sn-glycero-3-phospho-(1′-myo-inositol)
	M413T386	LIG	Alpha-d-mannose pentaacetate
	M482T190_2	LIG	1-hexadecyl-sn-glycero-3-phosphocholine
	M500T27	LIG	Oleyloxyethylphosphorylcholine
Negative mode	M124T601	HIG	N-(2-furoyl)glycine
	M157T603	HIG	2-isopropylmalic acid
	M169T36	HIG	Gallic acid
	M321T22_1	HIG	Deoxythymidine 5′-phosphate (dTMP)
	M429T49	HIG	(1-acetyloxy-3-hydroxy-6,8a-dimethyl-7-oxo-3-propan-2-yl-2,3a,4,8-tetrahydro-1h-azulen-4-yl) 4-hydroxybenzoate
	M482T77	HIG	Taurolithocholic acid sulfate
	M688T146	HIG	Pe 32:1
	M139T29	LIG	Chelidonic acid
	M275T29	LIG	Methyl salicylate
	M279T65	LIG	12s-hydroxy-5z,8e,10e-heptadecatrienoic acid
	M295T154	LIG	C10-dats (tentative)
	M319T45	LIG	20-HETE
	M329T36	LIG	(z)-9,12,13-trihydroxyoctadec-15-enoic acid
	M339T154	LIG	Cilastatin

HIG, chickens with high intramuscular fat content; LIG, chickens with low intramuscular fat content.

In negative mode, the levels of 14 metabolites displayed significant differences between the two groups. In which, seven metabolites were enriched in HIG, including N-(2-furoyl)glycine, 2-isopropylmalic acid, gallic acid, dTMP, (1-acetyloxy-3-hydroxy-6,8a-dimethyl-7-oxo-3-propan-2-yl-2,3a,4,8-tetrahydro-1h-azulen-4-yl) 4-hydroxybenzoate, and Pe 32:1. Significantly, taurolithocholic acid sulfate (a type of bile acids) was also enriched in HIG (Figure 5D and Table 3). The relationships between these five IMF-related bile acids were explored and were found that they were closely positively correlated with each other (Figure 5E). Seven metabolites were enriched in LIG, including chelidonic acid, methyl salicylate, 12s-hydroxy-5z,8e,10e-heptadecatrienoic acid, C10-dats (tentative), 20-HETE, (z)-9,12,13-trihydroxyoctadec-15-enoic acid, and cilastatin.

We next performed metabolic pathway analysis (MetPA) on the differential metabolites to explore the possible metabolic pathways associated with IMF of chickens. The results showed that these differential metabolites were mainly enriched in Riboflavin metabolism, Histidine metabolism, Pyrimidine metabolism, and Primary bile acid biosynthesis (Figure 5F), indicating that changes in these metabolic functional pathways may affect the deposition of intramuscular fat in chickens.

### Correlation of differential cecal microbiota and differential serum metabolites

**Figure 6:**
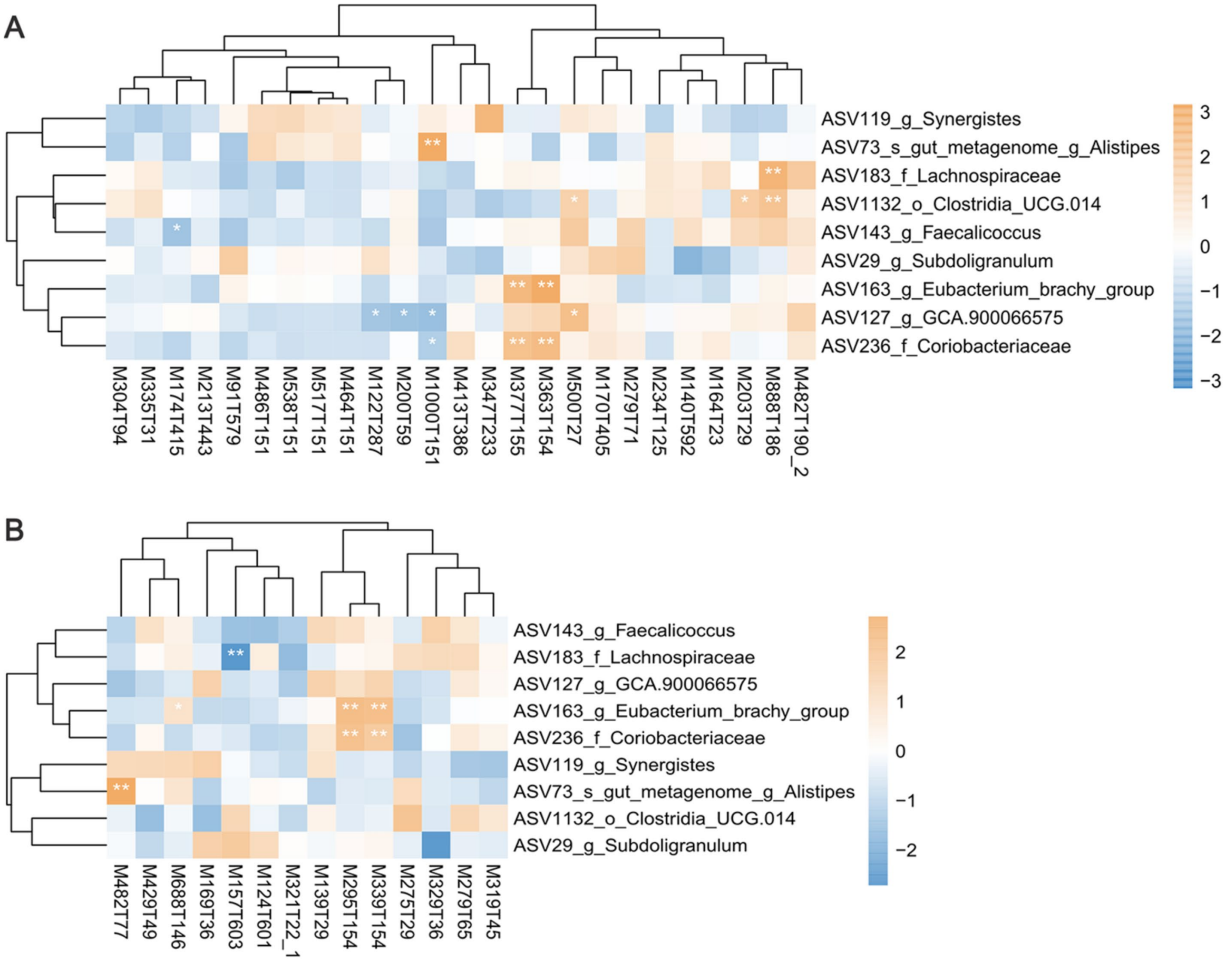
The correlation analysis between differential bacteria and differential serum metabolites. Heatmaps showing the correlations between differential bacteria and differential serum metabolites in **(A)** positive mode and **(B)** negative mode. Positive correlations are displayed in brown and negative correlations in blue. ^*^p < 0.05, ^**^p < 0.01, and ^***^p < 0.001.

A Spearman correlation analysis was conducted to establish the relationship between cecal microbiota and the metabolites (Figure 6 and Supplementary Table S6). As mentioned above, bile acids are important metabolites enriched in the serum of HIG chickens. The *Alistipes* (ASV73) enriched in the HIG were significantly positively correlated with the bile acids of taurolithocholic acid sulfate (M482T77, *r* = 0.409, *p* = 0.003) and taurochenodeoxycholic acid (M1000T151, *r* = 0.408, *p* = 0.003), while the *GCA-900066575* (ASV127) enriched in the LIG were significantly negatively correlated with taurochenodeoxycholic acid (M1000T151, *r* = −0.335, *p* = 0.016). This suggests that *Alistipes* may promote bile acids synthesis and thus regulate fat deposition in muscle, while *GCA-900066575* may degrade bile acids and reduce IMF in chickens. *Unclassified_f_Coriobacteriaceae* (ASV236, *r* = 0.387, *p* = 0.049) and *Eubacterium_brachy_*group (ASV163, *r* = 0.565, *p* < 0.001) enriched in LIG were significantly positively correlated with riboflavin (M377T155), glufosinate (M363T154), C10-dats (tentative) (M295T154), and cilastatin (M339T154). It indicates that these two bacteria may promote the synthesis of riboflavin and other metabolites, which is not conducive to fat deposition in chicken muscle.

## Discussion

Intramuscular fat content is a key factor affecting the tenderness and flavor of chicken (Hocquette et al., 2010). Gut microbiota have been found to affect the deposition of intramuscular fat by regulating the expression of genes and proteins related to fat synthesis and decomposition through the production of short-chain fatty acids, bile acids and other metabolites (Nicolucci et al., 2017; Parseus et al., 2017). To investigate the role of gut microbiota in intramuscular fat formation in chickens, we integrated the data of cecal microbiota and metabolome to explore the relationships among cecal microbiota, serum metabolites and IMF in chickens. The results showed that bile acids may be key metabolites that promote fat deposition in chicken muscle, and specific cecal bacteria could affect the IMF content of breast muscle via regulating the circulating levels of bile acids, riboflavin and other metabolites.

An important goal of this study was to identify key cecal bacteria associated with IMF of chicken. Previous reports had reported that the gut bacteria such as *Olsenella*, *Slackia* (Xiang et al., 2021), *Methanobrevibacter* (Wen et al., 2019), *Lachnoclostridium*, *Christensenellaceae_R-7_*group (Lei et al., 2022) were beneficial to abdominal fat deposition in chickens. However, various studies had failed to reach a consistent conclusion. *Methanobrevibacter* was the only commonality between two studies (Wen et al., 2019;Xiang et al., 2021) and was believed that can promote accumulation of abdominal fat by enhancing energy capture (Xiang et al., 2021). As mentioned above, the composition and formation mechanisms of intramuscular fat and abdominal fat differ (Zerehdaran et al., 2004). A recent study showed that low-abundance of *vadinBE97* was related to higher IMF of chicken muscle (Wen et al., 2023). In our study, *Synergistes*, *Subdoligranulum*, and *Alistipes* were enriched in HIG, while *unclassified_f_Lachnospiraceae*, *GCA-900066575*, *norank_f_norank_o_Clostridia_UCG-014*, *Eubacterium_brachy_*group, *Faecalicoccus*, and *unclassified_f_Coriobacteriaceae* were enriched in LIG. This inconsistency might be caused by the redundancy of gut microbiota functions. In the gut microbiome, functional redundancy is a ubiquitous phenomenon and microbial ecosystem functions could be independent of species content (Louca et al., 2018). Functional redundancy was the basis for stability and resilience (resistance to perturbation) of microbial ecosystems (Tian et al., 2020). This might indicate that taxonomically different bacteria in the ceca of different breed chickens performed similar metabolic functions and roles in IMF deposition. Among the cecal microbiota enriched in the HIG chickens, *Alistipes* and *Synergistes* belong to Bacteroidetes at the phylum level, which are involved in intestinal carbohydrate fermentation, utilization of nitrogenous substances and biotransformation of bile acids (Tamana et al., 2021). *Subdolicapsulum* is a butyrate-producing bacterium (Holmstrom et al., 2004). Among the cecal microbiota enriched in the LIG chickens, *GCA-900066575* belongs to the Lachnospiraceae family, which hydrolyzes starch and other sugars to produce butyrate and other short-chain fatty acids, and they have considerable ability to utilize dietary polysaccharides (Vacca et al., 2020). *Eubacterium* is an important butyrate-producing bacterium that plays an important role in regulating inflammation, modulating immune responses, maintaining intestinal barrier integrity, and cholesterol homeostasis (Mukherjee et al., 2020). *Faecalicoccus acidiformans* isolated from the chicken cecum, and reclassification of *Streptococcus pleomorphus*, its function has not been reported yet (Barnes et al., 1977). The functions of these bacteria may directly or indirectly promote the deposition or decomposition of fat in chicken muscles, which was partially confirmed by the correlation analysis between bacteria and metabolites in this study. However, their mechanism of action remains to be further studied.

Gut microbiota of chickens can be effectively intervened by changing feed ingredients, adding probiotics, or performing fecal microbiota transplantation, thereby improving the meat quality of chickens. For example, adding probiotics to chicken feed can significantly improve its meat color (Zheng et al., 2014) and flavor characteristics (Wang et al., 2017). What’s more, fecal microbiota transplantation allows transfer of the propensity for adipogenesis and the properties of muscle from donors to recipients (Lei et al., 2022). However, there are few reports on dietary intervention studies targeting intramuscular fat content in chickens. Some studies on other agricultural animals have found that dietary intervention can regulate the IMF content of the host by changing the gut microbiota. For example, it has been found that tylosin treated piglets shows changed composition of gut microbiota, up-regulation of gene expression related to fatty acid uptake and *de novo* synthesis in the longissimus dorsi muscle, and down-regulated gene expression related to triglyceride hydrolysis, which in turn increases the content of intramuscular fat in the longissimus dorsi muscle (Yan et al., 2020). *Prevotella* is considered to be closely related to the intramuscular fat content of pigs. Chen et al. (2021) administered *Prevotella* obtained from pig feces samples into mice and found that it significantly increased the IMF content of pigs. Transplantation of gut microbiota from Laiwu pigs, a Chinese pig breed with high intramuscular fat content, to Duroc × Landrace × Yorkshire pigs increases the expression of genes and proteins related to lipid synthesis in the muscles of recipient pigs, in turn significantly increases their IMF content (Xie et al., 2022).

In the differential metabolites, bile acids, Pe 32:1 and other metabolites were enriched in the serum of high-IMF chickens while riboflavin, some amino acids and other metabolites were enriched in the serum of low-IMF chickens. Pe 32:1 is a phospholipid of IMF components (Smith et al., 1998) and suggests that high levels of circulating Pe 32:1 was an important factor affecting IMF in chickens. Riboflavin is essential for mammalian growth and its derivatives flavin mononucleotide (FMN) and flavin adenine dinucleotide (FAD) are key co-enzymes in cells (Rudzki et al., 2021). Chickens with low IMF might still be in a growing state and had higher demand for vitamins and this would account for higher levels of riboflavin. The results of correlation analysis showed that *Eubacterium_brachy_*group was positively correlated with riboflavin, and the interaction of these might promote growth and have a negative regulatory effect on IMF formation.

It is worth noting that five bile acids of taurodeoxycholic acid, taurochenodeoxycholic acid, taourusodeoxycholic acid and taurohyodeoxycholic acid and taurolithocholic acid sulfate were all enriched in the sera of high-IMF chickens and displayed significant correlations with each other. Therefore, we speculated that these bile acids form a metabolic pathway and synergistically promote deposition of IMF in chickens. The pathway enrichment analysis indicated that primary bile acid biosynthesis was an important differential pathway related to IMF and verified our speculation. Primary bile acids are synthesized from cholesterol and can be combined with taurine or glycine in the liver. They are normally stored in the gallbladder and released into the duodenum after eating to help emulsify fat (Ridlon and Hylemon, 2012). Bile acids play roles as signaling molecules to regulate the digestion and absorption of cholesterol, triglycerides and fat-soluble vitamins (de Vos et al., 2022). The gut microbiota can convert primary bile acids (cholic and chenodeoxycholic acids) into secondary bile acids (deoxycholic acid and lithocholic acid) (Urdaneta and Casadesus, 2017) that then can activate nuclear receptors leading to upregulation of genes involved in adipocyte differentiation and adipogenesis and promote fat accumulation (Shinohara and Fujimori, 2020). Bacteria from the phyla Firmicutes, Actinobacteria, and Bacteroidetes can encode bile salt hydrolases (Jones et al., 2008). In our study, *Alistipes* (belong to Bacteroidetes) and *GCA-900066575* (belong to Firmicutes) were both found to have important associations with bile acid metabolism. *Alistipes* was enriched in HIG and displayed a significant positive correlation with taurolithocholic acid sulfate and taurochenodeoxycholic acid. This implicated *Alistipes* in the conversion of these bile acids that would activate nuclear receptors to result in enhanced IMF deposition. Studies in dairy cows have confirmed that bile salt hydrolase gene carried by *Alistipes* can hydrolyze conjugated bile salts into free bile acids and drive the conversion of bile acids (Lin et al., 2023). The *GCA-900066575* enriched in the LIG were significantly negatively correlated with taurochenodeoxycholic acid, suggesting that *GCA-900066575* may promote the degradation of bile acids, which is not conducive to the deposition of fat in muscles and leads to lower IMF content in LIG chickens. Liu et al. (2022) also found in human studies that GCA-900066575 is closely related to a variety of BAs, but its mechanism of action has not yet been clarified.

## Conclusion

The IMF content of breast muscle of Guizhou local chickens ranged from 1.65 to 4.59%. The complexity and stability of cecal microbiota network of low-IMF chickens were higher than those of high-IMF chickens. Bile acids may be important serum metabolites affecting IMF in chickens. Specific bacteria including *Alistipes* might promote deposition of IMF in chickens via bile acids and other metabolites, while the *Eubacterium_brachy* group and *Coriobacteriaceae* promoted formation of riboflavin, Glufosinate, C10-dats (tentative), and Cilastatin and were not conducive to the IMF deposition.

## Data Availability

The datasets presented in this study can be found in online repositories. The names of the repository/repositories and accession number(s) can be found in the article/Supplementary material.

## Footnotes

^1^ https://www.metaboanalyst.ca

^2^ http://huttenhower.sph.harvard.edu/galaxy
