# Supplementary material for: The chicken cecal microbiome alters bile acids and riboflavin metabolism that correlate with intramuscular fat content
Source: Front Microbiol. 2024 Dec 10;15:1494139. doi: 10.3389/fmicb.2024.1494139 (PMC11667789; doi:10.3389/fmicb.2024.1494139)
Supplement: Supplementary file 1 [file Table_1.DOCX]

| **Supplementary Table 1 Composition and nutrient level for chickens in this study(air-dried basis)** | | |
| --- | --- | --- |
| Item | 0-6 weeks | 6-18 weeks |
| ingredients，% |  |  |
| Corn | 56.30 | 58.62 |
| Soybean meal | 18.52 | 25.00 |
| Rapeseed meal | 10.00 | 0.00 |
| Corn gluten meal | 6.33 | 3.05 |
| Wheat bran | 2.94 | 5.63 |
| Soybean oil | 1.63 | 3.34 |
| Limestone | 1.18 | 1.18 |
| Phytase | 0.04 | 0.00 |
| Choline chloride | 0.15 | 0.00 |
| Methionine | 0.15 | 0.10 |
| Lysine | 0.22 | 0.32 |
| NaCl | 0.15 | 0.15 |
| CaHPO_4_ | 1.89 | 1.61 |
| Premix^1^ | 0.05 | 1.00 |
| total | 100.00 | 100.00 |
| Nutrients^2^, % |  |  |
| CP | 21.18 | 19.05 |
| ME,MJ/kg | 12.12 | 12.56 |
| Ca | 1.0 | 0.90 |
| AP | 0.45 | 0.40 |
| Met+Cys | 0.90 | 0.72 |
| Lys | 1.06 | 0.90 |
| ^1^Provides per kg of diet:  0-6 weeks: Vitamin A,6 000 IU; Vitamin B_1_, 2.0 mg; Vitamin B_2_, 4.0 mg; Vitamin B5, 42 mg; Vitamin B_6_, 4. 0 mg; Vitamin B_12_, 0.0l mg; Vitamin D_3_, 2 000 IU; Vitamin E 30 IU; Vitamin K3,1.8 mg; calcium pantothen-ate,10.0 mg; biotin, 0.15 mg; folic acid, 0.85 mg; Fe, 80 mg; Cu, 8.0 mg; Mn, 80 mg; Zn, 65 mg; I, 0.50 mg; Se, 0.25 mg.  6-18 weeks: Vitamin A,10 000 IU; Vitamin B_1_, 2.50 mg; VB_2_, 7.5 mg; Vitamin B_6_, 3 mg; Vitamin D, 2 000 IU; Vitamin E, 25 mg; Vitamin K, 2.8 mg; nicotinamide 40 mg; calcium pantothenate, 25 mg; biotin 0.20 mg; folic acid 1.5 mg; VB_12_, 0.015 mg, Fe, 80 mg;Cu, 8 mg , Mn, 100 mg; Zn, 60 mg; I , 0.35 mg; Se 0.3 mg.  ^2^ CP was a measured value, while the others were calculated values. | | |
